# High-Sensitivity Room-Temperature Power Sensor Based on a Graphene Oxide–PDMS Bilayer and Surface Plasmon Resonance Suitable for the Detection of IR-THz Radiation

**DOI:** 10.3390/s26134263

**Published:** 2026-07-04

**Authors:** Giancarlo Margheri, Tommaso del Rosso

**Affiliations:** 1 Institute for Complex Systems, National Council of Researches of Italy, Via Madonna del Piano 10, 50019 Sesto Fiorentino, Italy; 2 Departamento de Fisica, Pontifical Universidade Catolica, Rio Predio Cardeal Lema, Rua Marques de Saqo Vicente, 225, Gavea, Rio de Janeiro 22451-900, Brazil; tommaso@puc-rio.br

**Keywords:** plasmonic sensors, terahertz and infrared detection, thermo-optical devices

## Abstract

The accurate detection and quantification of electromagnetic radiation in the infrared (IR) and terahertz (THz) regions are critical for modern applications, yet they remain challenging due to the “THz gap” and the limitations of current room-temperature technologies. This paper proposes a novel uncooled IR–THz power sensor based on a hybrid graphene oxide (GO) and polydimethylsiloxane (PDMS) bilayer integrated into a surface plasmon resonance (SPR) architecture in the Kretschmann configuration. The device exploits the broadband optical absorption of GO to efficiently convert incident radiation into heat, while the high thermo-optic coefficient of the PDMS layer translates these thermal variations into measurable refractive index shifts. Finite Element Method (FEM) modeling was employed to optimize the sensor design, predicting a linear angular shift of 0.093 deg/mW. Experimental results confirm the theoretical expectations, demonstrating a high sensitivity of 0.083 deg/mW and an exceptionally low limit of detection and resolution on the order of 15 nW. By eliminating the need for cryogenic cooling or vacuum packaging, this platform offers a compact, low-cost, and high-performance solution for next-generation IR–THz metrology.

## 1. Introduction

Accurate detection of infrared (IR) and terahertz (THz) radiation is increasingly important for applications ranging from non-destructive inspection and biomedical diagnostics to spectroscopy and next-generation wireless communications [1,2,3,4]. However, the so-called “THz gap” continues to limit the availability of compact, sensitive, and cost-effective detectors operating at room temperature. Existing IR–THz detectors can be broadly classified into photon-based and thermal detectors. Photon detectors generally provide high sensitivity and fast response but often require cryogenic cooling to suppress thermal noise. Thermal detectors, including bolometers, pyroelectric sensors, and thermopiles, operate at room temperature and offer broadband spectral response, although their sensitivity is frequently limited by thermal losses, environmental fluctuations, or the need for vacuum packaging and mechanical modulation [5,6,7,8]. Recent efforts have focused on combining efficient photothermal absorbers with highly sensitive optical transduction mechanisms [9,10]. Graphene oxide (GO) is particularly attractive in this context owing to its broadband absorption—from the visible to the THz range [11,12]—low cost, solution processability, and compatibility with flexible substrates.

Graphene-based materials have recently attracted considerable attention in IR–THz photodetection due to their exceptional optical, electrical, and thermal properties. Several studies have demonstrated how graphene and its derivatives can be engineered to enhance photothermal conversion, mechanical robustness, and environmental stability. Examples include crumpled graphene field-effect transistors with efficient light-to-heat transduction [13], oxidation-resistant graphene coatings for long-term stability under high-power illumination [14], and multiband tunable THz refractive index sensors based on graphene plasmonics [15]. Within this broader context, GO represents an attractive choice for thermal detectors because it combines broadband absorption with solution processability, low cost, and strong adhesion to elastomers such as PDMS. These features make GO an ideal absorber for hybrid thermo-optic architectures such as the GO–PDMS–SPR platform proposed in this work. In parallel, polydimethylsiloxane (PDMS) exhibits one of the largest thermo-optic coefficients among polymeric materials, enabling efficient conversion of temperature variations into refractive index changes. Surface plasmon resonance (SPR), which is intrinsically sensitive to refractive index perturbations, provides an effective optical readout mechanism for such thermo-optic effects.

**Figure 1:**
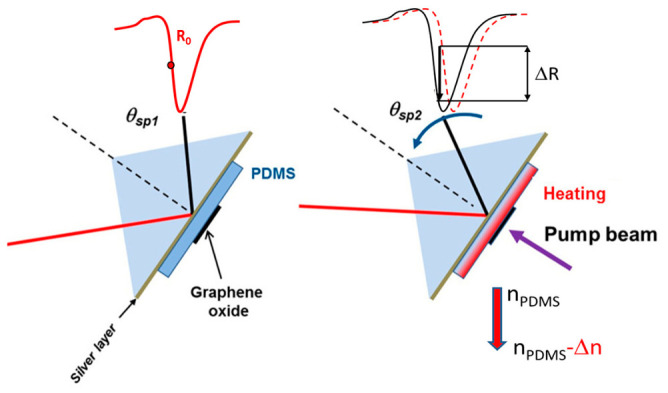
Principle of measurement. (**Left**): A GO-PDMS-Ag plasmonic multilayer exhibits an SPR spectrum with a minimum reflectivity at the angle θ_sp1_. When GO absorbs heating radiation (**right**), the elastomer refractive index lowers, the SPR spectrum shifts to a lower resonance angle θ_sp2_ and the reflectivity jumps from R_0_ to R_0_ − ΔR.

In this work, we propose and experimentally demonstrate an uncooled GO–PDMS–Ag/SPR power sensor for IR–THz metrology. The device combines broadband photothermal conversion in GO with thermo-optic transduction in PDMS and SPR interrogation in a Kretschmann configuration. Finite element modeling is used to optimize the sensor architecture and predict its response. Experimental characterization demonstrates a sensitivity of 0.083 deg mW^−1^ and a limit of detection of approximately 15 nW at room temperature, without vacuum packaging or cryogenic cooling. These results position the proposed platform among the most sensitive room-temperature thermo-optic detectors reported to date and highlight its potential for compact and low-cost IR–THz sensing applications. The working principle is schematically represented in Figure 1. The GO layer acts as an efficient broadband absorber, while the PDMS layer converts the resulting temperature rise into a refractive index shift that is detected by the SPR system. This hybrid GO–PDMS–Ag/SPR structure enables the measurement of minute temperature variations under standard laboratory conditions while remaining compatible with solution-processed fabrication and low-cost materials. This hybrid GO-PDMS-Ag/SPR device enables the measurement of minute temperature variations, even with standard lab conditions, while remaining compatible with solution-processed fabrication and low-cost materials. The resulting platform constitutes a promising route toward next-generation IR–THz metrology devices by combining broadband optical absorption, high thermo-optic sensitivity, and robust optical readout.

Moreover, thanks to the extremely wide absorption band of GO, the thermal response of the device can be evaluated without resorting to expensive IR–THz sources. This calibration strategy is consistent with commercial THz thermal detectors. For instance, the Gentec-EO THz5I-BL-BNC (Gentec EO, Quebec, QC, Canada) and the Ophir 3A-P-THz (Ophir Optronic Solutions Ltd., Jerusalem, Israel) are devices that are factory-calibrated using visible or near-infrared lasers. The same rationale applies to the GO–PDMS–SPR sensor, where the 405 nm pump serves as a reliable surrogate for THz illumination. Although the absorption coefficient of GO in the THz region is lower than in the visible range, the relatively large thickness of the deposited GO multilayer (~1.7 μm) is expected to provide significant THz absorption. Literature values indicate absorption coefficients on the order of 10^2^–10^3^ cm^−1^ at 0.1 THz and 10^3^–10^4^ cm^−1^ around 10 THz, corresponding to estimated absorbed fractions ranging from ~15% to >80% for the GO thickness used in this work. Therefore, the photothermal conversion mechanism is also expected to remain effective under THz illumination, although direct experimental validation with a THz source will be the subject of future dedicated investigations. The intrinsically high sensitivity of the SPR interrogation scheme, combined with the efficient heat generation and thermo-optic response of the GO–PDMS bilayer, enables the device to operate over a measurement range up to 50 mW with a limit of detection of 15 nW, placing this platform among the most sensitive room-temperature thermal detectors currently available.

## 2. Finite Element Modeling

**Figure 2:**
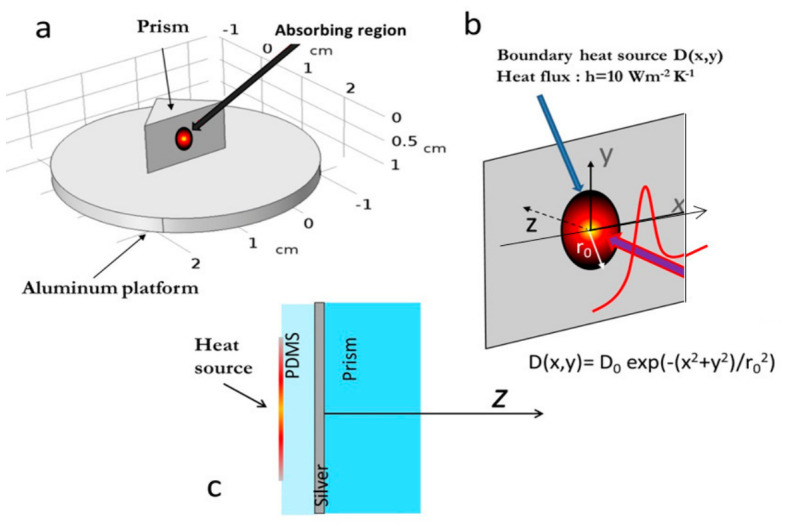
Representation of the SPR coupling system used in the Finite Element Modeling. (**a**) Assembly of the sensing platform constituted by the rotating base and the SPR multilayer on the prism. (**b**) Gaussian light intensity distribution on the heated area; D_0_ = 10.2 mW/mm^2^; 2∙r_0_ = 0.25 mm. (**c**) Domain of calculation for the temperature and refractive index distribution.

To predict the thermal behavior of the sensor, a three-dimensional finite element model was developed. The simulation focuses on the photothermal conversion occurring in the GO layer and on the subsequent heat transfer through the PDMS elastomer. The thermal analysis was performed using COMSOL Multiphysics (version 5.3). The aim of the modeling is to determine the refractive index variation of PDMS as a function of the optical power absorbed by the GO layer. The geometry used for the thermal analysis is shown in Figure 2. The system consists of an SF4 glass roof prism whose hypotenuse is coated with a 50 nm thick silver film. A PDMS layer with thickness t ranging from 0 to 100 μm, in steps of 25 μm, is placed on top of the silver layer. The external PDMS surface is covered with a circular GO layer with a diameter of 500 μm, which acts as the optical power absorber. The pump beam is focused into a circular spot with a uniform intensity distribution and a diameter of 250 μm. The boundary conditions used in the FEM analysis are illustrated in Figure 2. The pump beam is modeled as a Gaussian beam with a peak power density D_0_ = P_h_/πr_0_^2^, where Ph is the heating power, set to 1 mW unless otherwise specified.

Briefly, the heat generated by the light absorption diffuses partly in the elastomer and is partly transported by convection towards the external medium (air). We will consider a conservative situation in which weak air currents are present close to the device, and in this case, we can assume a typical convection coefficient of 10 Wm^−2^K^−1^, valid for vertical surfaces. The other physical parameters used for the analysis are directly taken from the database of COMSOL.

### 2.1. Convective Boundary Condition

Heat exchange with the surrounding air was modeled through a natural convection boundary condition applied to the external surfaces of the sensor. A convection coefficient of h = 10 W m^−2^ K^−1^ was adopted, which lies within the commonly accepted range for free convection in air, namely, 5–25 W m^−2^ K^−1^ for vertical surfaces under ambient laboratory conditions [16]. This value is widely used in thermal modeling of microsystems and polymer-based photothermal devices and provides a realistic approximation of heat dissipation in the absence of forced airflow. Sensitivity analyses performed by varying h within the typical free-convection range showed only minor variations of ≈5% in the predicted temperature rise, confirming the robustness of the model assumptions.

### 2.2. Thermo-Optic Coefficient of PDMS

The thermo-optic response of PDMS was described using a thermo-optic coefficient (TOC), dn/dT = −4.5·10^−4^ K^−1^, reported in the literature for cured Sylgard 184 in the near-infrared (NIR) spectral range with minor variations in the whole NIR range [17]. The large negative thermo-optic coefficient of PDMS represents the dominant mechanism responsible for the SPR resonance shift induced by photothermal heating. Variations in dn/dT reported in the literature are typically within ±10%, depending on curing conditions, wavelength, and cross-linking ratio, and therefore do not significantly affect the predicted sensor performance.

A ±10% variation in the thermo-optic coefficient and a ±50% variation in the convection coefficient resulted in less than a 8% variation in the predicted angular sensitivity, indicating that the model is weakly sensitive to uncertainties in these parameters.

### 2.3. GO Absorption

The GO layer thickness (~1.7 μm, as reported in the Section 4.2 is several times larger than the characteristic absorption depth reported for graphene-derived materials in the investigated spectral range. Assuming Beer–Lambert attenuation, where even conservative values of the absorption coefficient of ∼104–105 cm^−1^ lead to attenuation lengths ranging from 0.1 to 1 μm, a 1.7 μm thick GO film absorbs more than 85–99% of the incident radiation, depending on wavelength. Under these conditions, treating the absorbed power as uniformly converted into heat represents a reasonable first-order approximation for the thermal analysis. The small value of the GO layer thickness justifies the choice of a Boundary Heat Source as the boundary condition for the thermal analysis.

### 2.4. Methods, Approaches and Convergence of the Numerical Model

To ensure the numerical reliability of the finite element simulations, a systematic mesh convergence analysis was carried out in COMSOL Multiphysics. The computational domain was discretized using a non-uniform tetrahedral mesh with progressive local refinement in the regions exhibiting the highest thermal gradients, namely, the GO absorber, the upper PDMS surface, and the Ag–PDMS interface. Three mesh levels were considered: coarse mesh: ~1.2 × 10^5^ elements, with a minimum element size of 2.5 µm in PDMS; intermediate mesh: ~3.8 × 10^5^ elements, with a minimum element size of 1.0 µm; and fine mesh: ~1.1 × 10^6^ elements, with a minimum element size of 0.4 µm in the GO/PDMS region. For a representative heating power of P_h_ = 10 mW, corresponding to a theoretical surface temperature rise of ΔT ≈ 20 °C, the following values of the maximum external temperature increase ΔT_ext_ were obtained: 19.42 °C, 19.87 °C, and 20.03 °C for the coarse, intermediate and fine meshing, respectively, with 0.7% relative variation between the fine and intermediate meshing cases. The corresponding relative variation in the refractive index was 0.7%. Since both key output quantities varied by less than 1%, the solution was considered mesh-independent. All simulations reported in this manuscript were therefore performed using the intermediate mesh, which provides an optimal balance between accuracy and computational cost. The validity of the numerical model was further assessed through sensitivity analyses and comparison with experimental measurements. The influence of the main physical parameters, including the PDMS thermo-optic coefficient and the air convection coefficient, was investigated by varying them within the ranges reported in the literature. The resulting changes in the predicted angular sensitivity remained below 8%, confirming the robustness of the model assumptions. Additional validation was obtained by comparing the FEM predictions with the experimental results. The simulated angular sensitivity of the SPR resonance was 0.093 deg/mW, while the experimentally measured value was 0.083 deg/mW, corresponding to a deviation of less than 11%. Similarly, the theoretical limit of detection (12 nW) was found to be in close agreement with the experimentally determined value (15 nW). Overall, the convergence analysis, parameter sensitivity study, and comparison with experimental data confirm the numerical stability and predictive capability of the proposed FEM model.

**Figure 3:**
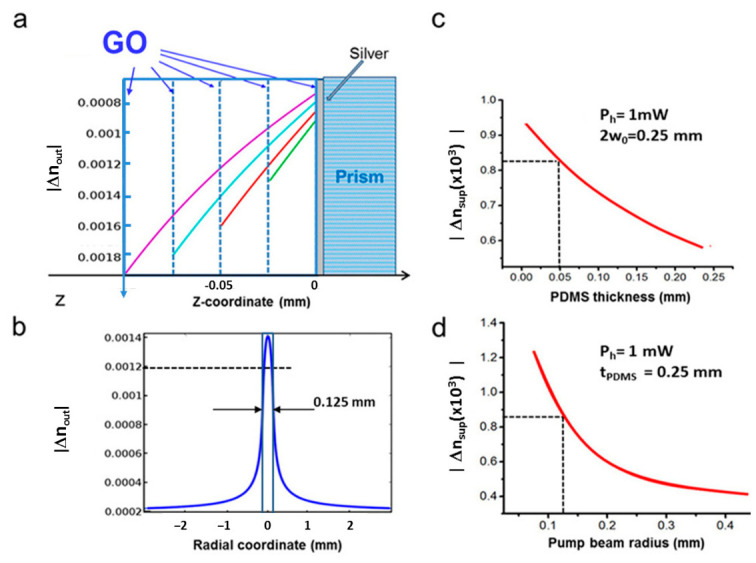
FEM study of the GO–PDMS–SPR sensing structure. (**a**) Simulated refractive index variation across the PDMS layer for different GO–metal distances. (**b**) Radial distribution of the thermo-optically induced refractive index change. (**c**) Dependence of the maximum refractive index variation on PDMS thickness. (**d**) Maximum refractive index variation vs. pump beam radius.

In Figure 3a, the z-variation in the refractive index of PDMS from the center of the hot area is reported for the elastomer thicknesses t = 0 mm, 0.025 mm, 0.05 mm, 0.075 mm and 0.1 mm.

As expected, the ratio Δn_sup_/Δn_out_ decreases significantly with increasing t, passing from 1 for very small thicknesses to 0.37 for t = 0.1 mm, with a rate of change of 6.3 RIU/mm.

Figure 3b shows the radial change in Δn_out_ at z = 0 mm. Considering an area around the axis with radius 0.125 mm (namely, the spot size of the focused pump beam), and the case of a PDMS thickness of 50 μm, namely, the thickness used in the experiments, the value of Δn_out_ ranges between the two close values of −1.2 × 10^−3^ and −1.4 × 10^−3^. Given this slight difference, for the following calculations, Δn_sup_ will be approximated with an average flat value of −1.3 × 10^−3^ for r < 0.125 mm and Δn_sup_ = 0 elsewhere.

The values of Δn_sup_ calculated at the surface between Ag and PDMS for several thicknesses of the elastomer are shown in Figure 3c. The variation decreases with increasing thickness in a quasilinear way, with an average rate of 1.4 RIU/mm.

Figure 3d reports the variation in Δn_sup_ versus the beam radius r_0_ of the heating pump beam. The radius increase corresponds to lower laser intensity, and it was calculated that doubling r_0_ corresponds to a decrease in |Δn_sup_| of approximately 40%, passing from 0.8 × 10^−4^ RIU to 0.5 × 10^−4^ RIU.

**Figure 4:**
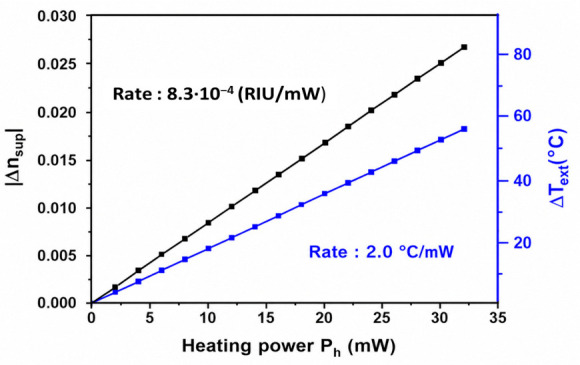
Ag–PDMS interface refractive index; the temperature change at the external PDMS surface vs. P_h_.

Figure 4 shows the change in the temperature ΔT_ext_ at the external PDMS–air interface and the value of Δn_sup_ at the Ag–PDMS vs. P_h_ interface. The behavior is linear in both cases, and their rate of change values are Δn_sup_/ΔP_h_ = 8.3 × 10^−4^ RIU/mW and ΔT_ext_/ΔP_h_ = 2.0 °C/mW. The threshold temperature of 100 °C is thus reached at P_h_ = 50 mW.

**Figure 5:**
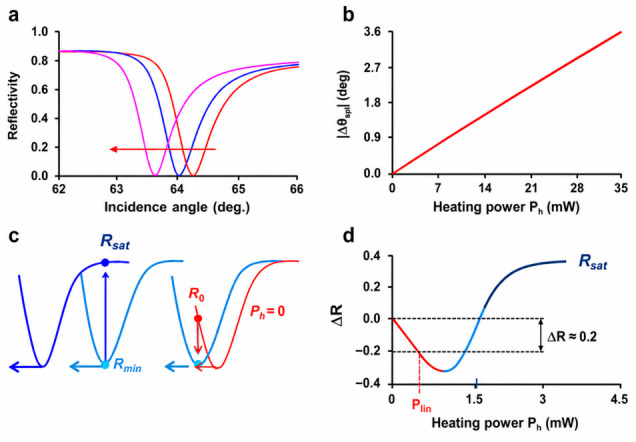
(**a**) Calculated SPR spectra for increasing P_h_ and (**b**) linear trend of the SPR angle shift vs. P_h_. (**c**) Sequence of the change in the reflectivity at a fixed angle from the base value R_0_ vs. P_h_ increase. The overall behavior is reported in (**d**). The trend can be considered as linear in the range of P_h_ from 0 to P_lin_ = 50 μW.

Then, the calculation of the shift in the SPR plasma angle θ_sp_ was carried out. The results are shown in Figure 5a, where the SPR angular spectra, that is, the plots of the reflectance R vs. the incidence angle θ, shift to the left almost conformally when P_h_ increases. The positions of their minima were calculated, with the results shown in Figure 5b. The plasma angle θ_sp_, whose initial value at P_h_ = 0 mW is 64.20 deg, varies linearly with P_h_, with an angular sensitivity of S_θ_ = Δθ_sp_/ΔP_h_ = 0.093 deg/mW. At the maximum theoretically allowed heating power of 50 mW, the left shift is thus 4.65 deg, which can be considered as the maximum theoretical angular range of the sensor.

Considering the angle of incidence θ of the left flexpoint of the SPR spectrum, where the sensitivity to the spectrum change is maximized, the corresponding reflectivity R_0_ (hereafter referred to as base reflectivity) decreases with increasing P_h_, as shown in Figure 5c, as a consequence of the left shift of the spectrum. The calculated variation ΔR= R − R_0_ is illustrated in the plot in Figure 5d for θ_0_ = 64.13 deg and R_0_ = 0.4, namely, the coordinates of the left flexpoint of the angular spectrum. A monotonic increase in P_h_ causes a decrease in the reflection R down to a minimum R_min_ = 2.3 × 10^−3^, found at P_h_ = 0.84 mW. For a higher P_h_, the reflectivity turns to grow to a saturation value of R ≈ 0.82. The trend of ΔR with P_h_ in the whole range is thus nonlinear, and in this case, the value of P_h_ can be suitably inferred from the measurement of Δθ_sp_ by exploiting the relationship Δθ_sp_/ΔP_h_ = S_θ_ = const. However, for heating powers below ≈ P_lin_ ≈ 50 μW, ΔR and P_h_ are almost proportional at fixed θ_0_, and this offers an easier tool to retrieve the value of P_h_ that does not require the time-consuming measurement of the angular spectrum to individuate the corresponding θ_sp_. More specifically, defining the reflectance sensitivity as S_R_ = d(ΔR)/dP_h_, calculated at P_h_ = 0 mW, we find that the modulus of S_R_ is equal to 0.112 mW^−1^ at θ_0_ = 64.13 deg, and this value remains constant within ±2.5% up to P_h_ = P_lin_. Once ΔR is measured, the corresponding P_h_ is readily found from the equation P_h_ = ΔR/S_R_. At incidence angles different from the inflection point, the sensitivity decreases. For example, at θ_0_ = 64.33°, i.e., 0.2° higher than the flexpoint, the magnitude of SR decreases by ≈40%, dropping to 0.067 mW^−1^, corresponding to a sensitivity loss of 200% per degree. This high value highlights the critical importance of precise prism positioning, which will be further discussed in the Section 4.1 Calibration is still possible for higher P_h_, but due to the nonlinear behavior of ΔR vs. Ph, more complex fitting functions are required, making the retrieval of Ph less straightforward. Finally, we provide a theoretical estimate of the minimum detectable heating power, i.e., the limit of detection (LOD). Assuming a refractive index resolution of 1 × 10^−8^ RIU, consistent with the best values reported in the literature [18], and using the theoretical result Δn_sup_/ΔP_h_ = 8.3 × 10^−4^ RIU/mW, the corresponding LOD for P_h_ is 12 nW. As shown in the Section 4, a similar value can be achieved experimentally, although longer averaging times are required to compensate for environmental instabilities. To summarize, this section has derived the main theoretical features of the sensor using the physical parameters listed in Table 1. These values represent reference parameters that may differ from those of the actual experimental setup; therefore, the modeling provides an approximation of the real behavior. Nevertheless, the theoretical predictions, summarized in Table 2, are shown to be in good agreement with the experimental results presented in the next section.

The sensitivity S_R_ = d(ΔR)/dP_h_ is computed at the angle θ = 64.15° of the left flexpoint of the SPR angular spectrum for P_h_ = 0 mW. See text for further details.

## 3. Experimental Details

### 3.1. Sample Fabrication

Commercial microscopy slides were cut into 25 × 25 mm squares, cleaned, and inserted into an evaporation chamber. The cleaning procedure consisted of a coarse wash with a commercial detergent, followed by rinsing with flowing water and consecutive ultrasonic baths in ethanol and trichloroethylene (20 min each). Pure silver (99.95% purity, Goodfellow Italia, S.r.L, Milano, Italy)) was evaporated using a Joule heater (Consolini Paolo, Parma, Italy) at a rate of approximately 50 Å/s, and the metal thickness was monitored in real time with a quartz crystal microbalance until the nominal 50 nm thickness was reached. The PDMS layer was fabricated using Sylgard 184 (Dow Italia. S.r.l., Milano, Italy.) A liquid mixture with a 10:1 monomer–curing agent ratio was prepared and degassed for approximately 30 min. As explained in Section 2, the polymer thickness must be as small as possible to efficiently transfer heat to the Ag interface. This was achieved by fabricating thin PDMS layers using the doctor blade technique [20]. Two strips of commercial 60 μm thick aluminum adhesive tape were placed parallel on the glass sample at a distance of about 1 cm. The width of the area between the strips matched the slide width (2 cm), giving an area A = 2 cm^2^. The assembly was weighed with an electronic balance (resolution 0.1 mg), yielding a mass M1. The liquid Sylgard mixture was then dispensed between the strips, and a razor blade was used to spread the material and remove excess fluid. The assembly was weighed again, yielding M2. Knowing the PDMS density (ρ = 1.03 g/cm^3^), the average thickness tav was calculated as: t_av_ = (M2 − M1)/(A·ρ). The sample was then placed on a heater at 50 °C for 10 min to initiate polymerization, followed by 24 h curing at room temperature. This method produced PDMS layers with thicknesses of approximately 55 μm, close to the nominal tape thickness. The absorption of the Ag–PDMS stack was measured using the pump laser Cobolt 06-MLD, 405 nm, 150 mW CW (Cobolt AB, Solna, Sweden). The incident power P_0_, reflected power P_R_, and transmitted power P_T_ were measured, and the absorption was calculated as A = (P_0_ − P_R_ − P_T_)/P_0_. As anticipated, the sensor measures the impinging power indirectly through the thermal variation in the PDMS refractive index. Therefore, the impinging power must be converted into heat with the highest possible efficiency. GO was obtained from commercial purified natural graphite (Cabro S.p.A. Arezzo, Italy) using the Hummers method [19], yielding grains with sub-micrometer lateral dimensions and a height of ~1.5 nm. A stock aqueous GO solution (100 mg/L) was prepared after synthesis. Then, 1 mL of the stock solution was concentrated tenfold by slow evaporation under weak vacuum, and 1 μL drops were sequentially deposited onto the Ag–PDMS surface and dried under ~10^−2^ Torr. A similar GO film was fabricated on a PDMS-only substrate to measure GO absorption. Transmission was measured by illuminating the GO layer with the pump laser and collecting both on-axis and scattered light with a sensor placed close to the sample. The drop-casting procedure was repeated until the transmitted power fell below 1% of the input power. Seven drops were required to reach this condition. Only one GO multilayer deposition was investigated, as the goal at this stage was to demonstrate the proof-of-concept operation of the GO–PDMS–SPR architecture rather than optimize the GO thickness.

### 3.2. Displacement of the Resonance Angle vs. P_h_

**Figure 6:**
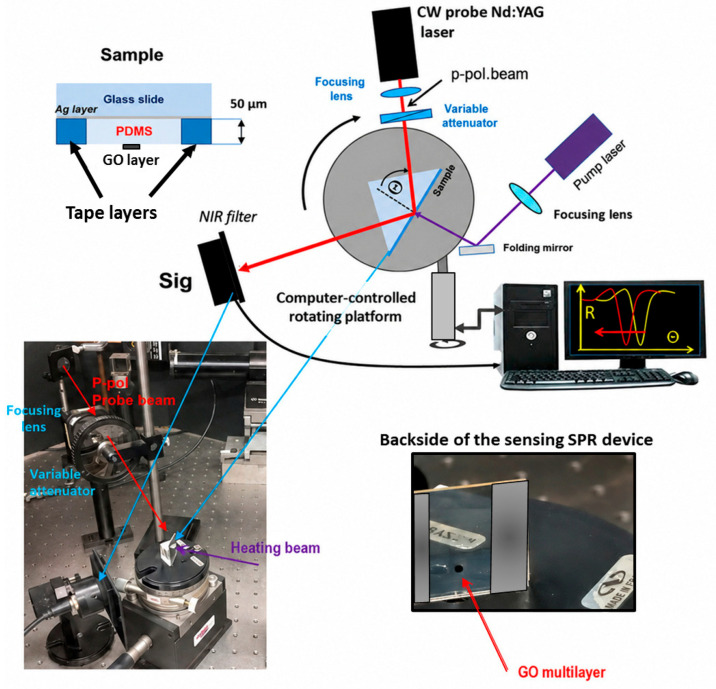
Experimental layout of the SPR layout used to measure the θ_sp_ vs. P_h_ variation. The reflected signal is NIR high-pass-filtered and measured by a signal detector (Sig). The resonance angle θ_sp_ is locked with a 0.002° precision. The photos show the main details of the bench hosting the experimental apparatus (down on the left) and a visualization of the fabricated sample coupled to the prism (down on the right).

A key objective of this work is to investigate the relationship between the SPR resonance angle θ_sp_ and the pump power Ph using the experimental layout shown in Figure 6. Before this measurement, the fabricated silver layer was characterized using the same setup. The Ag–PDMS–GO sample was oil-coupled to an SF4 glass roof prism in the Kretschmann configuration. The IR probe laser Roithnertechnik RLTMIL-1064-500-5, 1064 nm, 500 mW CW, (Roithner Laser Technik Gmbh, Wien, Austria) was attenuated to obtain an impinging power of 100 μW and slightly focused on the prism hypotenuse using a 40 cm focal length lens, producing a spot with a 390 μm diameter at 90% intensity, measured with an independent 10× optical system. The prism was rotated until the reflectivity reached its minimum at θ_sp_. The rotating platform was then scanned around this angle with 0.002° steps, and the angular spectrum was recorded.

In the following step, the heating laser beam was slightly focused using a lens with a focal length of 250 mm, producing a focal spot with a 90% intensity diameter of 350 μm (measured independently with the same magnifying optical system previously used for the probe beam characterization). The heating beam was then carefully superimposed onto the probe beam focus. The pump light was absorbed by the GO layer and converted into heat. As a consequence, the refractive index of the Sylgard layer decreased, and the resonance angle θ_sp_, which is highly sensitive to the external refractive index, shifted accordingly. In a first set of tests, we measured the variation in θ_sp_ as a function of P_h_. To this aim, we recorded the resonance spectra for a set of heating powers ranging from 0 mW to 22 mW, and the corresponding resonance angles were extracted from the best fit of the experimental curves. The theoretical external temperature rise ΔT_ext_ at P_h_ = 22 mW is approximately 52 °C (see Figure 4), which corresponds to an elastomer temperature of about 72 °C, well below the decomposition temperature of PDMS (≈250 °C). The output of these measurements is the calibration curve Δθ_sp_ vs. P_h_. The ΔR–P_h_ relationship was investigated using the setup shown in Figure 6, following the procedure described below. First, the optimal base angle θ_0_ had to be determined. A first-guess position θ_0,1,_ corresponding to a theoretical reflectivity of approximately 30%, was selected. The prism was then carefully moved back and forth using the rotation stage in steps of 0.005°, and the corresponding signal variation ΔR_1_ was recorded. The initial setpoint θ_0,1_ was then slightly shifted to a new position θ_0,2,_ and a new variation ΔR_2_ was measured. This procedure was iterated until ΔR was maximized at a specific angular position, which was then defined as the base angle θ_0_ and kept fixed for the subsequent measurements. Next, the sensing area on the prism hypotenuse was illuminated with increasing pump power. This produced a corresponding decrease ΔR in the reflectivity, and the trend ΔR vs. P_h_ was recorded until the relationship between P_h_ and ΔR deviated significantly from linearity. The best fit of the results allowed us to determine the calibration curve of the sensor in the low-power regime.

### 3.3. Measurement of LOD and Resolution

**Figure 7:**
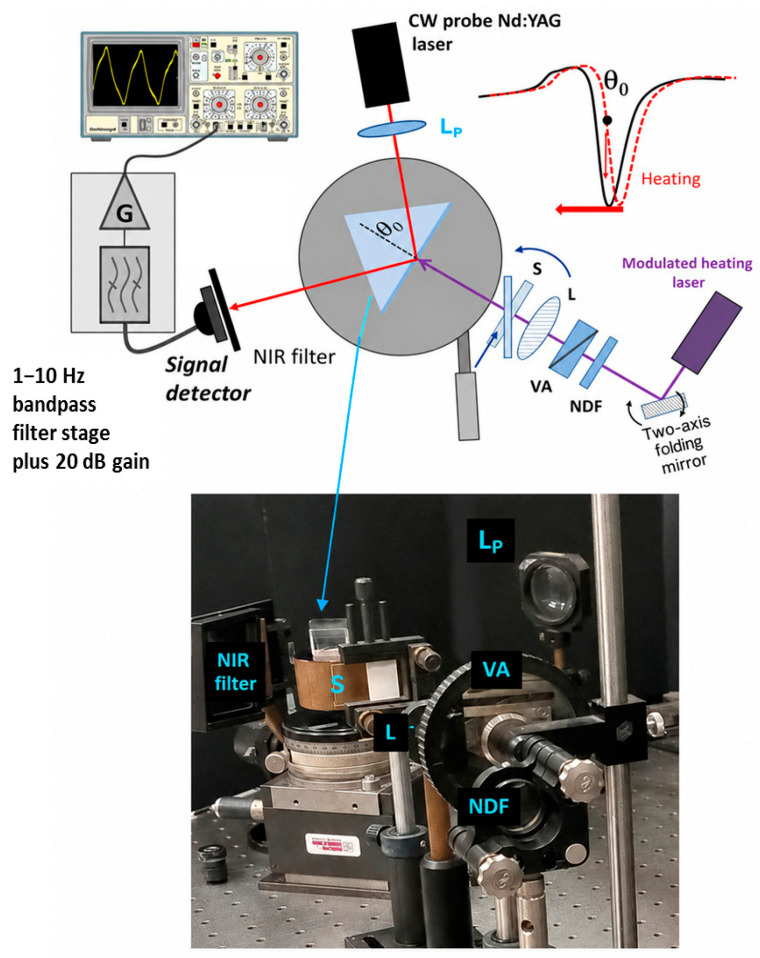
Measurement of LOD and resolution. VA: optical variable attenuator; NDF: neutral density filter chain (from OD = 3 to OD = 7.1). S: rotable glass slide, inserted to measure the resolution at P_h_ = 150 nW by exploiting the losses due to the Fresnel reflections; L: focusing lens. See text for further details. The photo shows some details of the bench hosting the experimental apparatus.

In the next experimental step, we examined the LOD of the sensor. As the aim of the tests is to measure very low heating powers, they were performed at a fixed angle, with the setup illustrated in Figure 7. The prism is thus placed at the incidence angle θ_0_ individuated in the Section 3.2.

The most effective strategy for monitoring very small variations in the probe signal is to modulate the pump laser beam at frequency f and detect the presence of a corresponding component in the Fourier spectrum of the probe signal. To this aim, the modulable heating laser was driven with a square wave signal generated by an external function generator, with a 50% duty cycle and manually adjustable frequency. The delivered power was set to its maximum value (150 mW) to ensure the highest emission stability. The periodic heating induces a periodic variation in the Sylgard refractive index, which, in turn, produces periodic fluctuations in the reflected probe signal ΔP_probe_ around a continuous level P_probe_. The reflected light was detected using a large-area Hamamatsu PIN photodiode with responsivity at 1064 nm equal to 0.07 A/W (Hamamatsu Photonics Italia, Arese, Italy) coupled to a Thorlabs PDA200C transimpedance amplifier (Thorlabs Gmbh, Newton, NJ, USA). The resulting voltage signal, composed of a continuous component V0 and a modulated component at frequency f, was sent to a Teledyne LeCroy T3DS001104 oscilloscope operating at 10 MSa/s (Teledyne Le Croy Inc., Chestnut Ridge, New York, NY, USA). The output signal was then filtered using a Wavetek 852 filter bench (Wavetek Corp., San Diego, CA, USA). Specifically, the filter chain consisted of a high-pass filter (cut-off frequency 1 Hz) followed by a low-pass filter (cutoff frequency 10 Hz), forming a passband filter with a 9 Hz bandwidth. This configuration effectively suppressed the continuous component V_0_, the low-frequency 1/f noise, and the parasitic 50 Hz interference from the laboratory electrical network. The interplay between heat generation, thermal dissipation, and subsequent electronic processing results in a frequency-dependent amplitude response, which can be readily verified by varying the modulation frequency. The oscilloscope FFT (Fast Fourier Transform) function was used to detect the presence of a modulated component at the driving frequency f. Using the optical attenuation chain, the pump power was progressively reduced until the minimum detectable variation above the noise floor was reached. Because environmental disturbances introduce random reflectivity fluctuations on the same order of magnitude as the effect under investigation, this measurement suffers from significant signal stability issues at low pump powers. This limitation was partially mitigated by averaging multiple acquisitions, at the cost of increased measurement time. The resolution of the system, defined as the minimum detectable change in power, was evaluated by setting the attenuation to 6 OD, corresponding to a heating power of 150 nW on the sample. This power was then slightly reduced until the corresponding change in the signal amplitude became clearly observable. The fine additional attenuation required was conveniently achieved by tilting a microscope coverslip placed in front of the prism hypotenuse, exploiting its angle-dependent transmission. This geometrical configuration was reproduced in a separate setup, which allowed the transmission of the tilted coverslip to be measured accurately.

### 3.4. Statistical Dispersion and Measurement Repeatability

To quantify the statistical reliability of the experimental results, each measurement was repeated five times under identical conditions. The angular scan measurements used to extract θ_sp_ were repeated after realignment of the prism, yielding a dispersion of the extracted resonance angle below ±3.5% over the full power range. The reflectivity-based measurements (ΔR vs. P_h_) showed a significantly lower variability, with a sensitivity dispersion of ±2.5%, consistent with the FEM-predicted linearity of the reflectivity slope at the flexpoint. For the LOD tests, the modulated probe signal amplitude was recorded over several modulation cycles, resulting in a fluctuation of ±8%, mainly attributable to environmental air convection noise and to the mechanical tolerances of the rotation stage. Overall, the observed dispersion is low and fully compatible with the theoretical predictions of the thermal model, confirming the robustness and reproducibility of the sensing mechanism.

## 4. Results and Discussion

### 4.1. Plasmonic Response

**Figure 8:**
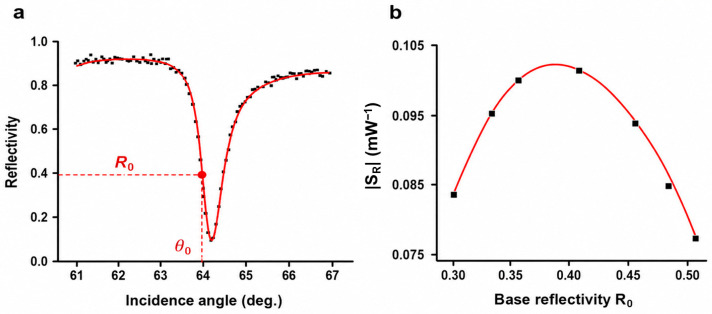
(**a**) A typical experimental SPR spectrum and its best fit (red line). The best fit provides the silver refractive index n_Ag_ = 0.041 + 7.17i, and the thickness of the layer is 56.3 nm. (**b**) Points: |S_R_| measured at base reflectivity R_0_. Red line: cubic fitting of the analytical form A + BR + CR^2^ + DR^3^ in which the numerical values of the coefficients are A = −3, B = 21.46, C = − 41.7, and D = 23.8.

The experimental angular spectrum of the Ag-PDMS bilayer is shown in Figure 8a. Assuming a refractive index of 1.41 for PDMS, its best fit provides the values of the refractive index n_Ag_ and thickness of the silver layer, namely, n_Ag_ = 0.041 + 7.17i, precise to ± 2.2% in both real and imaginary parts, and (56.3 ± 0.5) nm. The reflectivity minimum is R_min_ = 0.082. The optimization of the reflectivity minimum down to ≈2 × 10^−3^ would require a thickness of 63 nm with the same refractive index. The flexpoint angle of the best-fit spectrum results is θ_0_ = (63.94° ± 0.01°), with a corresponding reflectivity R_0_ = (0.382 ± 0.004).

**Figure 9:**
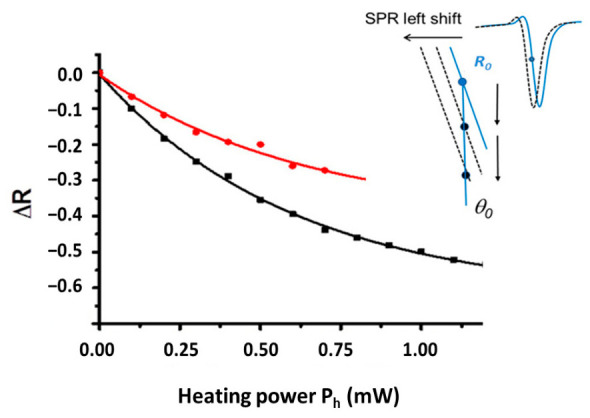
Decay in the reflectivity with P_h_ at different base reflectivities. Black dots: the base reflectivity is R_0_= 0.38 at the left flexpoint of the SPR spectrum. The best-fit line expression is ΔR = 0.62∙(e^−1.64∙Ph^ − 1), (P_h_ in mW). Red dots: base reflectivity R_0_ = 0.28. The best-fit line expression is ΔR = 0.41∙(e^−1.56∙Ph^ − 1). The inset is a cartoon of the decline in the reflectivity consequent to PDMS heating, as recorded at a fixed incidence angle θ_0_.

The sensitivity S_R_ = d(ΔR)/dP_h_ decreases in magnitude at incidence angles different from the flexpoint of the reflectivity spectrum. This was verified by measuring S_R_ at various incidence angles, corresponding to different values of R_0_. The results reported in Figure 9 show that S_R_ actually reaches its maximum magnitude, S_Rmax_ = 0.107 mW^−1^ (close to the theoretical prediction of 0.112 mW^−1^), at the angle θ_0_ = 63.94°.

The behavior of S_R_ was further checked by measuring the decrease in ΔR vs. P_h_ at θ_0_ = 63.94° and an angle 2° apart, namely, θ_1_ = 61.94°, associated with R_1_ = 0.2. The variation in S_R_ is clearly evidenced in the plots in Figure 9 as the variation in the slope of the experimental point distributions. The best fit of the curves gives a slightly different value for S_Rmax_ that passes from the measured 0.107 mW^−1^ for P_h_ close to zero mW to S_Rmax_ = 0.102 mW^−1^, while at R_0_ = 0.2, from the best fit, it is quite lower, namely, S_Rmax_ = 0.065 mW^−1^. Both evaluations are affected by uncertainties on the order of 0.3%. As previously anticipated, this finding suggests a very low tolerance to the positioning of the prism. Indeed, a simple calculation shows that a tolerance of the sensitivity S_Rmax_ of 5% requires positioning the angle θ_0_ with precision on the order of 0.02°.

It is worth noting that from the analytical best-fit expression (reported in the caption of Figure 9), one can calculate that S_Rmax_ remains constant within ±3.5% if the P_h_ range extends to 50 μW. This linearity is nicely aligned with the theoretically found value of ≈±2.5%.

**Figure 10:**
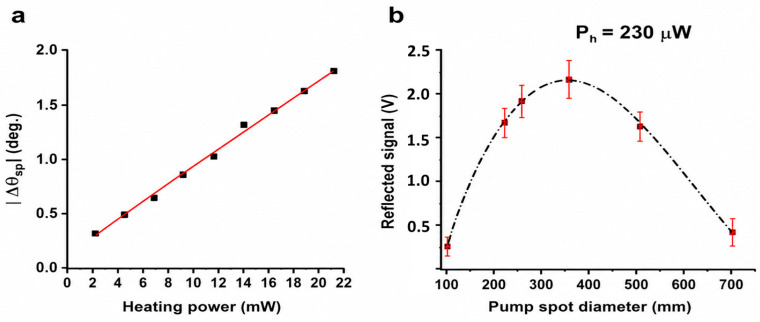
(**a**) Experimental shift in the resonance angle vs. the heating power P_h_ and best-fit red line, whose slope is 0.083 deg/mW. (**b**) SPR signal vs. the pump spot diameter. The probe spot diameter is 390 μm. The dash–dot line is only a guide for the eye.

The change in the resonance angle Δθ_sp_ vs. P_h_ is reported in Figure 10a for P_h_ ranging up to 22 mW. Although the theoretical highest power can be pushed to 50 mW, here, we preferred to stay well within the nominal safe range. However, we will check the remaining P_h_ range in a further experimental study, where the temperature rise will also be carefully monitored to guarantee a safe experimental operation.

The trend is linear, with an angular sensitivity whose modulus is 0.083 degmW, in good agreement with the theoretical value (0.093 deg/mW). The best fit of the experimental point distribution has an uncertainty of ±2.5%, meaning that this method allows measuring P_h_ with a full-scale precision (0–22 mW) of ±0.5 mW.

In the next step, we tested the tolerance of the sensing system to the geometrical mismatch between the probe spot (fixed diameter: 390 μm) and the variable diameter of the heating laser spot. Indeed, in the presence of a too-large pump spot, the pumping power is not completely used to heat the interrogation area. However, due to the high diffusion of heat in the GO layer, we expect that the geometrical mismatch may be mitigated, and the tolerance to this detrimental effect is expected not to be too tight as well. The experiment was performed by setting the incidence angle to θ_0_ = 63.94° and recording the voltage signal change while changing the pump spot diameter by using lenses with different focal lengths and optimizing the signal by moving the lens placed on a x-y-z translational stage. The results are shown in Figure 10b, which shows a decrease in the signal of 15% when the focal spot of the beam passes from the initial value of 350 μm to either 300 μm or 500 μm, corresponding to a tolerance range of −14% +43% with respect to the pump focal spot diameter, around 350 μm, which corresponds to the maximum signal. The overall tolerance, 57%, demonstrates that the system does not suffer significant response fall as far as the heating source diameter variation is within ≈±50 μm (±14%).

### 4.2. Characterization of the GO Absorbing Layer

**Figure 11:**
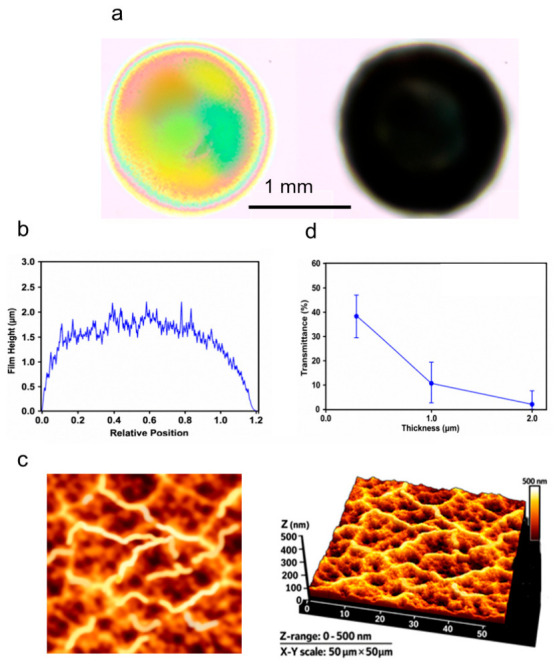
Characterization of the GO multilayer. (**a**) Optical microscope images of dried GO drops: 1 drop (left) and 7 drops (right). (**b**) Profile of the median scan of the essiccated drop. (**c**) AFM images of a 50 μm × 50 μm area of the GO layer. (Left): top view; (right): 3D AFM image. (**d**) Optical transmittance of the essiccated multilayer vs. the number of drops.

The GO layer was first characterized optically by conventional optical microscopy (microscope Novex Holland, Euromex Microscopes BV, Arnehm, The Netherlands). Figure 11a shows the difference in optical transmission between a deposition obtained by essicating one drop (left), while the effect of the essiccation of seven drops is shown on the right of the figure. In this second case, the almost complete extinction of the illumination light is evident. Then, we proceeded to the analysis with profilometry by using a Tencor Alpha Stepper 200 profilometer (KLA Corporation, Milpitas, CA, USA). Figure 11b shows the profilometric thickness profile taken on a median line of the resulting coating, revealing an average film thickness of approximately 1.7 μm across the sensing area. Surface morphology was also investigated by atomic force microscopy by using a Nanosurf Easyscan AFM (Nanosurf AG, Liestal, Switzerland) over a 50 × 50 μm^2^ region (Figure 11c), revealing a continuous multilayer structure characterized by interconnected wrinkles and corrugations typical of solution-processed GO films [21]. Analysis of the AFM topography indicates an average wrinkle height of approximately 285 nm, while the overall surface height variation extends over the 0–500 nm range. Such morphological features are commonly attributed to solvent evaporation, flake stacking, and residual stress relaxation occurring during the drop-casting process. The observed morphology is in good agreement with previous studies on multilayer graphene oxide coatings prepared by drop-casting [22], where stacked GO sheets form micrometer-scale corrugated networks with characteristic wrinkle amplitudes of a few hundred nanometers. Similar topographical features and multilayer arrangements have been reported in AFM investigations of drop-cast GO films employed in optical, thermal, and photothermal applications [23,24]. Optical transmission measurements performed at 405 nm (Figure 11d) showed a strong thickness-dependent attenuation, with the transmittance decreasing from approximately 38% for thinner coatings to about 2–3% for the 1.7 μm thick film. This corresponds to an attenuation exceeding 97%, confirming the strong absorbing nature of the multilayer GO coating. The measured optical behavior is consistent with the progressive accumulation of overlapping GO sheets and agrees with previously reported absorption characteristics of drop-cast GO multilayers [25]. Raman spectroscopy was not performed in the present study because the objective was not the structural characterization of graphene oxide itself but the evaluation of its performance as a broadband photothermal absorber within the SPR sensing architecture. Nevertheless, the measured thickness, AFM morphology, average wrinkle height (~285 nm), and optical attenuation are fully consistent with the characteristics reported for multilayer GO films fabricated by drop-casting, thereby supporting the validity of the absorber model adopted in the FEM simulation.

### 4.3. LOD and Resolution

**Figure 12:**
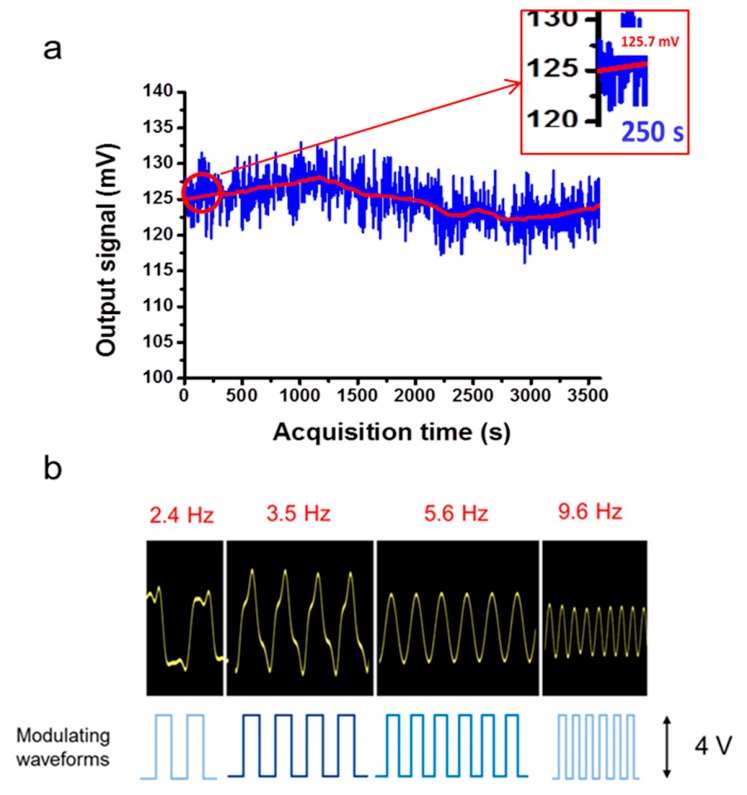
(**a**) Stability of the signal reflected from the prism at the setpoint R_0_. (**b**) Oscilloscope traces of the system response V_out_ to a square wave modulated input, amplitude 4 V, at different frequencies (lower part). The shape of the output signal changes with frequency, as well as its amplitude, reaching its maximum at approximately 3.5 Hz.

The final part of the experimental work was dedicated to evaluating the LOD and the resolution in the measurement of Ph. Since temporal stability is a key prerequisite, the stability of the GO–PDMS–SPR sensor was assessed over a one-hour continuous measurement at a fixed interrogation angle. As shown in Figure 12a, the normalized signal remained confined within a ±3.8% envelope, with an RMS fluctuation of approximately 0.35% and a long-term drift of about 4%. This level of stability is fully compatible with the laser specifications provided by the manufacturer (stability < 5%) and with the averaging times used for low-power measurements, confirming that the averaged thermo-optic response of the PDMS layer is not significantly affected by environmental fluctuations over extended periods. The observed noise floor is therefore dominated by intrinsic thermal fluctuations rather than systematic drift, supporting the robustness of the platform for continuous IR–THz power monitoring. Owing to this stability, the use of a reference measurement was deemed unnecessary. Moreover, as reported in the inset of Figure 12a, the average stability over 250 s is on the order of 0.6%. This enabled the detection of ultralow heating powers using averaging times of only a few tens of seconds. Because the expected power changes are very small, the prism must be positioned at the angle of maximum sensitivity. In addition, since a time-dependent variation in the output signal is expected—together with a frequency at which the response is maximized, mainly as a consequence of the electronic band-pass filter chain—we analyzed the temporal behavior of the sensor by setting the attenuation chain to OD = 3, thereby reducing the heating power to 1.5 mW to avoid overheating effects. As shown in Figure 12, the expected changes in both the shape and amplitude of the detected signal Vout were clearly observed. From these measurements, we experimentally identified the frequency fopt that maximizes the amplitude of V_out_, which was found to be approximately f_opt_ ≈ 3.5 Hz.

**Figure 13:**
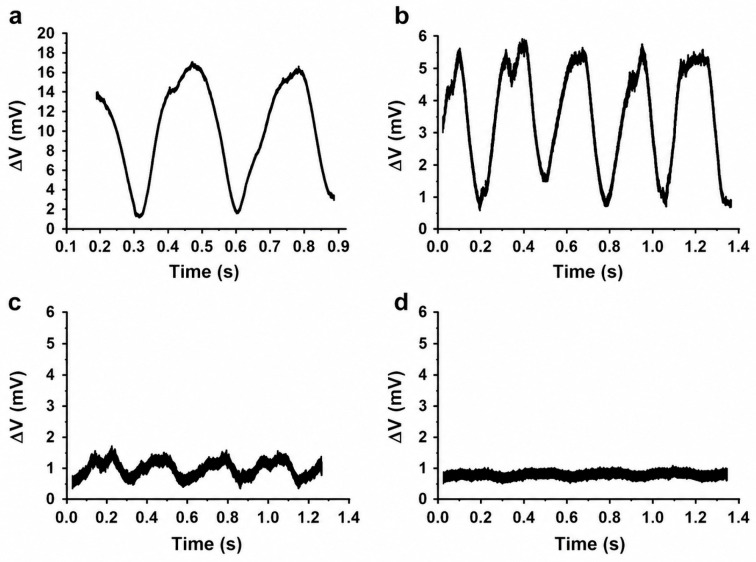
Measurement of the LOD of P_h_. Output signals for heating powers of (**a**) 150 nW (OD = 6), (**b**) 60 nW (OD = 6.4), (**c**) 15.0 nW (OD = 7.0), and (**d**) 10 nW.

**Figure 14:**
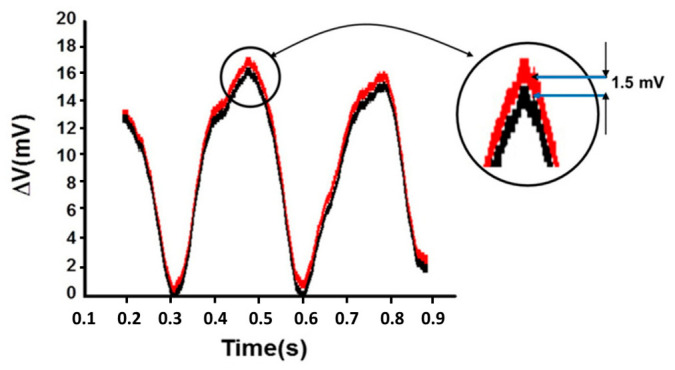
Measurement of the sensor resolution. Red line: Experimental trace of the signal output at P_h_ = 150 nW. The black line corresponds to P_h_ = 138 nW. Inset: Enlarged view of the top region of the signals, corresponding to P_h_ = 16.8 nW. The resolution estimated from the noise band is 11.2 nW.

Then, OD = 6 was chosen to obtain a P_h_ amplitude of 150 nW, with the result shown in Figure 13a. The signal obtained is well detected, and the voltage maxima, averaged on 16 acquisitions in a typical time of 3 s, have a value of V_av_ = 16.7 mV. Defining the voltage sensitivity S_V_ of the sensor as S_V_ = V_av_/P_h_, this results in S_V_ = 0.11 mV/nW. When the optical pump is attenuated with a further filter with OD = 0.4, lowering P_h_ to 60 nW, environmental instabilities cause the signal to become noticeably unstable (Figure 13b), making it necessary to average 32 acquisitions to improve its quality. In practice, this corresponds to a measurement time of approximately 7 s for a single value of P_h_. The standard deviation σ_V_, directly measured by the oscilloscope in AVERAGE mode, was σ_V_ = 0.42 mV, compared with an average maximum signal amplitude of 4.9 mV. Following an established criterion, the minimum detectable pump power is calculated as P_h,min_ = 3σ_V_/S_V_, yielding P_h,min_ = 11.5 nW. This result was experimentally verified by manually increasing the attenuation with the variable attenuator while simultaneously checking the presence of the modulated signal in the FFT. As expected, additional attenuation degraded the signal quality, increasing the averaging time required for a stable measurement to approximately 35 s. When the attenuation reached a sufficiently high value, the FFT no longer showed the 3.5 Hz component. Just before this condition was reached, the corresponding additional optical density was measured (0.6 OD), giving an overall attenuation of OD = 7.0, which reduced the heating power to P_h_ = 15 nW. This power produced the output signal shown in Figure 13c, where the 3.5 Hz modulation is still clearly visible and remains detectable in the FFT. At higher attenuation levels (Figure 13d), the modulation becomes almost indistinguishable, a condition confirmed by the FFT, which shows only random fluctuations around 3.5 Hz. Considering the calculated rate of change of the surface refractive index Δn_sup_/ΔP_h_ = 8.3 × 10^−4^ RIU/mW, the limit of detection of approximately 15 nW corresponds to a refractive index change of Δn_sup_ = 1.3 × 10^−8^ RIU, which is among the best reported values for refractive index resolution in an SPR apparatus. The resolution was measured following the procedure described in Section 3.3. The signals were recorded as averages of 64 acquisitions, providing sufficient stability of the peak-to-valley (P–V) values. The amplitudes corresponding to P_h_ = 150 nW and P_h_ = 138 nW are shown in Figure 14, together with the difference between the maxima of the central portion of the output modulation. As shown, the difference of 1.5 mV remains visible above the noise level, estimated as approximately 1.2 mV (3σ), corresponding to 11.2 nW. We conclude that the resolution is of the same order of magnitude as the LOD of the sensor.

The experimental results are summarized in Table 3, compared to the values calculated in the modeling section, showing their good agreement. The restriction of the measurement range has been previously justified. The overall measurement uncertainty was evaluated by considering the main sources of experimental error affecting the SPR-based power measurements, namely, the angular resolution of the optical interrogation system, fluctuations in the incident optical power, and environmental thermal perturbations. The angular position of the SPR minimum was determined through polynomial fitting of the resonance curve. Five repeated measurements performed under identical experimental conditions yielded a standard deviation of approximately 1.2 × 10^−3^ deg in the retrieved resonance angle. This value was taken as the angular uncertainty of the measurement system. The uncertainty associated with the incident optical power was estimated from the specifications and repeatability of the calibrated power meter used during the experiments. Over the investigated power range, the corresponding uncertainty remained below 1.5% of the measured value. Environmental temperature fluctuations represented the dominant noise source at low power levels. To quantify their contribution, five consecutive measurements were acquired over extended time intervals while maintaining constant illumination conditions. The resulting baseline fluctuations increased significantly for averaging times shorter than 20 s, whereas averaging periods between 25 and 35 s provided stable measurements close to the detection limit. The minimum detectable power was determined by propagating the angular uncertainty through the experimentally measured sensitivity according to ΔP = Δθ/S, where Δθ is the angular uncertainty and S is the sensor sensitivity. Using the measured sensitivity of 0.083 deg mW^−1^, the resulting power resolution was approximately 11–15 nW, in agreement with the experimentally observed LOD. The good agreement between repeated measurements, theoretical predictions, and experimental results confirms the reliability of the adopted measurement methodology and indicates that the residual uncertainty is mainly governed by environmental thermal stability rather than by the intrinsic performance of the GO–PDMS–SPR sensing architecture. To further assess the reliability of the proposed sensing platform, repeated measurements were performed under identical operating conditions over five experimental sessions. The SPR resonance curves exhibited highly consistent shapes and resonance positions, indicating stable optical and thermal behavior of the GO–PDMS–SPR structure throughout the measurement campaign. The measured sensitivity remained within the experimental uncertainty of the system (approximately 1.5% of the measured value) and showed good agreement with the numerical predictions. In particular, the experimental sensitivity (0.083 deg mW^−1^) differed by less than 11% from the FEM prediction (0.093 deg mW^−1^), demonstrating the robustness of both the device architecture and the adopted modeling assumptions. The observed variability was primarily associated with environmental temperature fluctuations and long-term thermal drift of the laboratory environment rather than with irreversible changes in the sensing structure. No measurable degradation of the GO absorber, the PDMS thermo-optic layer, or the silver plasmonic film was observed during the experimental campaign. These results indicate good measurement repeatability and suggest that the dominant limitation to reproducibility at nanowatt power levels is external thermal stability rather than intrinsic device variability.

## 5. Discussion

The sensor operates in continuous-wave mode and remains stable up to several tens of milliwatts of incident power, providing a wide dynamic range while maintaining high sensitivity in the low-power regime. The measured refractive index resolution of approximately 1.3 × 10^−8^ RIU further confirms the excellent performance of the plasmonic readout architecture. A potential limitation of the proposed sensor is the averaging time required to achieve nanowatt-level detection. Although the intrinsic thermal time constant of the GO–PDMS–SPR structure is approximately 0.3 s, averaging periods of 25–35 s were necessary to suppress environmental thermal fluctuations and obtain stable measurements close to the detection limit. This requirement may appear restrictive when compared with fast thermal detectors such as microbolometers or pyroelectric sensors, whose response times are typically in the millisecond range. However, such devices are generally optimized for dynamic signal detection rather than for achieving the lowest possible detectable power under continuous-wave operation [26]. A more meaningful comparison can be made with highly sensitive room-temperature detectors operating under similar conditions. Golay cells, lock-in-based thermal detectors, and several thermo-optic sensing architectures frequently rely on signal averaging, modulation techniques, or extended integration times to reach nanowatt or sub-microwatt detection limits [27]. In these systems, the effective acquisition time is often determined not by the intrinsic detector response but by the signal-to-noise ratio required to resolve extremely small thermal perturbations. Therefore, the averaging time reported here should be regarded primarily as a consequence of the measurement conditions associated with ultralow-power detection rather than as an intrinsic limitation of the sensing architecture. For applications involving power monitoring, calibration, metrology, or laboratory THz measurements, acquisition times of a few tens of seconds are generally acceptable, particularly when accompanied by room-temperature operation, continuous-wave detection capability, and a detection limit in the tens-of-nanowatts range. Future developments aimed at improving thermal isolation and exploiting higher-Q plasmonic resonators [28] are expected to reduce the averaging time while preserving the excellent sensitivity of the platform. Beyond its sensing performance, the proposed GO–PDMS–SPR platform exhibits several characteristics that favor practical implementation. The fabrication process relies on low-cost materials and scalable techniques, including metal evaporation, doctor blade deposition of PDMS, and solution-based processing of graphene oxide, all of which are compatible with large-scale manufacturing. This suggests that the sensor architecture could be extended to array configurations or multiplexed sensing platforms without significant technological barriers. Several advantages also emerge when comparing our sensor with graphene-based devices. Graphene detectors typically require high-quality CVD growth, transfer onto the target substrate, encapsulation, and additional gating or biasing schemes. In contrast, our sensor is fully planar and does not rely on any 2D material processing, eliminating transfer-related defects and significantly reducing fabrication complexity. Moreover, graphene THz detectors often require external bias or operate under non-ambient conditions, whereas our device functions at room temperature with minimal or zero bias. The good agreement between FEM simulations and experimental results, together with the repeatability observed over multiple measurement sessions, indicates satisfactory reproducibility of both the fabrication process and the sensing mechanism. The main limitation currently arises from environmental thermal fluctuations and long-term drift, which become increasingly relevant when operating near the nanowatt detection limit. However, these effects are not intrinsic to the sensing architecture and could be mitigated through differential measurement schemes [29,30], reference channels [31,32], temperature stabilization, or active feedback control of the SPR operating point [33,34]. From an engineering perspective, the solid-state nature of the device offers advantages in terms of mechanical robustness and ease of integration compared with conventional Golay cells and other thermally sensitive detectors. Furthermore, the optical SPR readout can be readily interfaced with automated electronics and digital signal processing systems, enabling real-time monitoring and calibration functions. Finally, the broadband absorption of graphene oxide and the room-temperature operation of the platform make it attractive for integration into practical THz instrumentation, including power meters, source calibration units, spectroscopy setups, and emerging THz communication testbeds. These considerations suggest that the proposed architecture represents not only a high-sensitivity laboratory demonstrator but also a promising candidate for future compact and deployable THz sensing systems.

The comparison between the actual performances of the sensor provided in the present work and other state-of-the-art techniques is concisely shown in Table 4. The table shows that the GO–PDMS–SPR sensor achieves one of the lowest detection limits among room-temperature, uncooled IR–THz detectors, outperforming conventional pyroelectric and thermopile devices by more than one order of magnitude. Its performance is also comparable to that of MEMS microbolometers and Golay cells, while avoiding vacuum packaging and maintaining a simpler fabrication process. Although cryogenic bolometers remain unmatched in absolute sensitivity, their operational constraints make them unsuitable for compact, low-cost systems. In contrast, the GO–PDMS–SPR platform provides a unique combination of ultralow LOD, ambient condition operation, and moderate fabrication complexity, positioning it as a strong candidate for next-generation IR–THz metrology.

## 6. Conclusions

In this work, we designed, fabricated, and characterized an uncooled optothermal power sensor based on a GO–PDMS bilayer integrated with an SPR transduction platform for IR–THz applications. The combination of the broadband absorption properties of graphene oxide and the high thermo-optic coefficient of PDMS enables efficient room-temperature radiation detection, offering a promising approach for THz metrology without cryogenic cooling. The experimental characterization demonstrated a linear response up to 50 µW and stable operation up to 22 mW. The device achieved an angular sensitivity of 0.093 deg/mW, corresponding to a refractive index variation of 8.4 × 10^−4^ RIU/mW. Most importantly, the sensor exhibited a limit of detection and resolution of approximately 10 nW, together with an outstanding refractive index resolution of 1.3 × 10^−8^ RIU, placing it among the best-performing SPR-based sensing platforms. The intrinsic thermal response time was estimated to be about 0.3 s, although practical measurements at the lowest power levels are currently limited by environmental thermal drifts. Compared with established technologies, the proposed solid-state architecture offers advantages in terms of robustness, low noise, and simplified operation. Future improvements based on optimized optomechanical designs and more sensitive plasmonic structures, such as plasmonic waveguide resonators, could further enhance the temporal response and sensitivity. Overall, the GO–PDMS–SPR approach provides a scalable and cost-effective foundation for next-generation portable THz power sensors.

## Figures and Tables

**Table 1:** The physical parameters used for the FEM simulation.

Material	Manufacturer	Refractive Index @1064 nm	dn/dT @1064 nm (°C^−1^)	Thermal Conductivity (W m^−1^ °C^−1^)
PDMS (Sylgard 184)	Dow Inc., Milano, Italy	1.46	−4.5 × 10^−4^	0.16
SF4 Glass	Schott, Mainz, Germany	1.7289	1 × 10^−5^	0.9
Silver (99.95% purity)	Goodfellow, Milano, Italy	0.05 + 4i	(2.1 + 3.5i) × 10^−4^	429
Purified Natural Graphite	Cabro S.p.A., Arezzo, Italy	N.D.	N.D.	N.D.
Graphene Oxide	Ref. [19]	2.0 + 0.45i (@405 nm)	N.D.	5

**Table 2:** The theoretical features of the sensor.

Δn_sup_/ΔP_h_ (RIU/mW)	ΔT_ext_/ΔP_h_ (°C/mW)	S_θ_ (deg/mW)	S_R_ (mW^−1^)	LOD (nW)	Range (mW)
8.3 × 10^−4^	2.0	0.093	0.112	12	0–50

**Table 3:** Experimental performances of the proposed sensor. Lower row: theoretical values (see Section 2). S_V_ = V/P_h_ Voltage sensitivity.

S_q_ (deg/mW)	S_V_ (mV/mW)	S_R_ (mW^−1^)	LOD (nW)	t_m_ (s)	Range (mW)
0.083	0.11	0.102	15 nW	35	0–22
0.093	N.D.	0.112	12 nW	N.D.	0–50

**Table 4:** Results of the present work (first line) compared to relevant state-of-the-art room temperature devices.

Technology	Angular Sensitivity (deg mW^−1^)	Refletance Sensitivity (mW^−1^)	LOD/Resolution (nW)	Power Range (nW–mW)	Response Time (s)	Operating Conditions	Fabrication Complexity
GO-PDMS-SPR sensor (this work)	0.083	0.102	15/11	15–22	0.3 intrinsic; 25–35 averaging	RT, CW, no bias	Medium
Pyroelectric detector	—	—	50–100	100–100,000	0.01–0.1	RT, modulated source required	Low
Thermopile	—	—	1000–10,000	10,000–1,000,000	0.01–0.1	RT	Low
MEMS microbolometer	—	—	10–100	100–10,000	0.005–0.02	RT, often vacuum packaged	High
Golay cell	—	—	1–10	10–10,000	0.01–0.05	RT, mechanically sensitive	Medium-High
Graphene photothermoelectric detector	—	—	1–100	1–10,000	10^−9^–10^−6^	RT, antenna-coupled	High
Graphene FET/bolometric detector	—	—	0.1–10	1–1000	10^−6^–10^−3^	RT, bias and gate control required	Very High
Schottky diode THz detector	—	—	10–100	100–100,000	10^−9^–10^−6^	RT, direct electrical readout	Medium
CMOS THz detector	—	—	100–10,000	1000–100,000	10^−6^–10^−3^	RT, integrated electronics	Medium-High

Abbreviations: RT = room temperature; CW = continuous wave. Note: The GO–PDMS–SPR sensor achieves one of the lowest detection limits among uncooled room-temperature detectors while maintaining moderate fabrication complexity.

## Data Availability

The original contributions presented in this study are included in the article. Further inquiries can be directed to the corresponding author.
